# *In-ovo* supplementation with bovine milk osteopontin improves hatchability, chick quality, growth performance, and enhances intraepithelial lymphocyte populations in specific-pathogen-free layer chicks

**DOI:** 10.1016/j.psj.2026.106569

**Published:** 2026-02-02

**Authors:** Bikas R. Shah, Shuja Majeed, Nimra Khalid, Pankaj Arora, Khaled Abdelaziz, Ali Nazmi

**Affiliations:** a Department of Animal Sciences, The Ohio State University, Wooster, OH, USA; b Arla Foods Ingredients Group P/S, Arla Foods Ingredients, Viby J, Denmark; c Department of Animal and Veterinary Sciences, Clemson University, Clemson, SC, USA; d School of Health Research, Clemson University, Clemson, SC, USA; e Food for Health Discovery Theme, The Ohio State University, Columbus, OH, USA

**Keywords:** Chick quality, Gut health, Hatchability, Immunity, Osteopontin

## Abstract

To evaluate the effect of *in-ovo* supplementation of bovine milk-derived Osteopontin **(bmOPN)** in chicks, fertilized specific-pathogen-free eggs were randomly allocated into six treatment groups (*n* = 26 eggs/group) on embryonic day 18. The hatchability was collected from six independent experiments. Eggs in each treatment received a 200 μL *in-ovo* injection of PBS containing 0 mg, 0.1 mg, 1 mg, 10 mg, 25 mg, or 50 mg of bmOPN. On day of hatch **(DOH)**, hatchability and chick quality parameters were assessed. Body weights were recorded on DOH, 7, and 14 days of age. On day 7 of age, intestinal histomorphometric parameters, including villus height **(VH)**, crypt depth **(CD)**, and VH:CD ratio, were measured in the 0, 1, and 25 mg groups. Peripheral blood mononuclear cells **(PBMCs)** and intraepithelial lymphocytes **(IELs)** were isolated from 0 and 1 mg groups for flow cytometry analysis. Data were analyzed using Generalized Linear Mixed Model, one-way ANOVA, Kruskal-Wallis or Mann-Whitney U test followed by Tukey HSD/Dunn test. The hatchability increased by approximately 8% in the 1 mg and 25 mg groups compared to the control, reaching 93.6% and 93.9%, respectively. We observed dose-dependent decreases in chick length and residual yolk percentage, along with an increase in navel score up to 25 mg bmOPN. Hatched chick body weights increased slightly (1–2 g) in the 1, 10, and 25 mg groups, and by day 14, chicks in the 1 mg and 10 mg groups maintained higher body weights and body weight gains. On day 7, bmOPN administration increased the number of intestinal T-cell IELs (TCRαβ^+^ subsets and TCRγδ^+^), as well as non-T IELs (including iCD8α^+^ cells), while no changes were observed in peripheral blood mononuclear cells. These findings suggest that *in-ovo* supplementation of bmOPN enhances hatchability, chick quality, growth performance, and mucosal immune development. However, further studies are warranted to investigate the effects of bmOPN administered through different routes on intestinal function and immune responses during inflammation in both broiler and layer chickens.

## Introduction

Osteopontin **(OPN)** is a glycophosphoprotein encoded by the secreted phosphoprotein (Spp)-1 gene. It contains a highly conserved specific arginine-glycine-aspartate (RGD) integrin binding motif across vertebrates (Denhardt and Guo, 1993), which contributes to the structural stability and functional integrity of OPN protein. OPN plays significant roles in various physiological processes, encompassing bone remodeling (Staines et al., 2012), embryo implantation and gestation processes (Dunlap, et al., 2008; Johnson, et al., 2014), brain development (Jiang, et al., 2019), and immune responses (Ashkar, et al., 2000; Hur, et al., 2007; Lund, et al., 2013; Murugaiyan, et al., 2008; Schack, et al., 2009; Shinohara, et al., 2008). It is secreted into various bodily fluids, such as blood, milk, and cerebrospinal fluid (Denhardt and Guo, 1993; Jiang, Prell and Lönnerdal, 2019). In chickens, OPN is linked to improved calcium and phosphorus bioavailability (Shet, et al., 2018), production of OPN by eggshell gland during eggshell calcification in circadian fashion (Arazi, et al., 2009) and eggshell biomineralization (Le Roy, et al., 2021), and osteogenic differentiation (Rath and Durairaj, 2022).

In mouse intestinal epithelium, the innate CD8α **(iCD8α)** cell, a unique cell to intraepithelial lymphocytes **(IELs)**, expresses the highest mRNA OPN levels relative to other intestinal cells (Hur, Youssef, Haws, Zhang, Sobel and Steinman, 2007). Both iCD8α cells and OPN are critical for survival and proliferation of IEL populations and promoting intestinal homeostasis in mice and humans (Nazmi, et al., 2020, 2019; Van Kaer, et al., 2014). In addition, OPN maintains the proper expression of Foxp3 T regulatory cells in the intestine, promoting the protection against colitis in a T-cell adoptive transfer colitis model (Nazmi, Greer, Hoek, Piazuelo, Weitkamp and Olivares-Villagómez, 2020).

Besides its role in intestinal mucosal immunity, several studies have shown that OPN may have multiple functions within the gastrointestinal tract. It interacts with cell surface receptors like integrins and CD44, which are essential for cell adhesion and migration, a critical process in forming various cell layers and structures in the developing intestine, including the intestinal epithelium (Jiang, et al., 2023). Additionally, OPN can activate various signaling pathways that influence cell behavior, potentially affecting cell proliferation, differentiation, and survival during intestinal development (Jiang, Lo, Prell and Lönnerdal, 2023; Karasalih, et al., 2025; Schack, Lange, Kelsen, Agnholt, Christensen, Petersen and Sørensen, 2009).

OPN is considered an important factor for infant growth and development because it is one of the most abundant proteins in human milk (Christensen and Sørensen, 2016; Jiang and Lönnerdal, 2019; Schack, Lange, Kelsen, Agnholt, Christensen, Petersen and Sørensen, 2009). OPN constitutes about 2.1% of the total protein content in human milk, with significantly higher levels in colostrum than in mature milk (Schack, Lange, Kelsen, Agnholt, Christensen, Petersen and Sørensen, 2009). Additionally, plasma OPN concentrations in 3-month-old infants and in umbilical cord blood are 7-10 times higher than those in adults. The supplementation of bovine milk OPN **(bmOPN)** in infant formulas for newborn rhesus monkeys and preterm piglets has been shown to enhance intestinal development (Aasmul-Olsen, et al., 2021; Donovan, et al., 2014). Also, bmOPN stimulated the *in vitro* proliferation of human intestinal epithelial cells (Liu, et al., 2020). Together, these findings suggest a pivotal role for milk OPN supplementation in promoting intestinal development, growth, and overall health, particularly during early life. However, the potential of bmOPN as a feed additive for chickens and its effects on their development remain largely unexplored.

During the last three days of incubation, chick embryos undergo a significant and rapid increase in intestinal development, characterized by substantial morphological, cellular, and molecular changes (Uni, 2006). Given that the newly hatched chicks possess a functionally immature gastrointestinal tract, which must transition from yolk-based nutrition to a formulated feed diet, promoting early growth and development of the intestine in newly hatched chicks is crucial for optimizing the growth of commercial chickens. While various approaches have been explored to enhance intestinal development, modulate microbiome composition, boost immune responses, and support overall gut health is *in-ovo* supplementation of nutrients, such as amino acids, minerals, vitamins, and probiotics (Alizadeh, et al., 2022; Jha, et al., 2019), little is known about the bmOPN in this context. Therefore, the primary objective of this study was to determine the impact of *in-ovo* administration of bmOPN on intestinal development, growth performance, and mucosal immune cell populations in hatched chicks.

## Materials and methods

All animals and animal protocols used in this experiment were approved by the Institutional Animal Care and Use Committee **(IACUC)** at The Ohio State University **(OSU)** protocol # 2021A00000010.

### In-ovo treatments and hatchability

Fertile specific-pathogen-free **(SPF)** White Leghorn chicken’s eggs were obtained from the OSU Centre for Food Animal Health (CFAH), The OSU, Wooster, OH, USA. The SPF flock is routinely monitored and certified as SPF according to CFAH health monitoring testing protocol by the OSU IACUC and Institutional Biosafety Committees (IBCs). Eggs of the same batch were collected freshly from an aged-matched SPF flock, and incubated in incubator (NatureForm Hatchery Systems, Model No. NMC 1620 Inc, FL, USA) at Poultry Research Unit at the OSU. On embryonic day (**ED**) 18, eggs were candled, sorted, and randomly allocated into six treatment groups (*n* = 26 eggs/group). The fertile eggs on each incubator tray were thoroughly mixed and then assigned to the six treatments by systematic randomization. Eggs in each treatment group were administered *in-ovo* with varying concentrations of bmOPN (Lacprodan® OPN-10, Arla Food Ingredients Group, Denmark) dissolved in 200 µL PBS as follows: 0, 0.1, 1, 10, 25, or 50 mg of bmOPN. The *in-ovo* injection delivered 1 inch depth into the amniotic fluid, using a 1.5-inch, 21-gauge needle as per the previously described protocol (Nazmi, et al., 2017). Then, eggs were transferred to the hatcher (NatureForm Hatchery Systems, Model No. NMC 1620 HAT, FL, USA). On the day of hatch **(DOH)**, hatchability of fertile eggs was calculated for each treatment as the number of chicks hatched / number of *in-ovo*-injected fertile eggs × 100. The whole experiment was repeated six times solely to obtain additional hatchability data, with each individual experiment (batch) treated as a block during statistical analysis (Block = 6). Only chicks hatched from the first incubated batch were raised for the collection of other parameters and samples.

### Housing, chick quality, body weight, and body weight gain

Hatched chicks of bmOPN treatment groups (0 mg, *n* = 23; 0.1 mg, *n* = 22; 1 mg, *n* = 25; 10 mg, *n* = 24; 25 mg, *n* = 25; and 50 mg, *n* = 21) were wing-tagged, placed in separate floor pens (1.2 × 1.2 m) within the same environmentally control room. Pens were bedded with fresh pine-wood shavings, and chicks had *ad libitum* access to feed and water. The lighting program consisted of 24 h light for the first week, followed by 23 h light: 1 h dark in the second week. On the DOH, chick quality was assessed, including chick length and naval condition from all the hatched chicks. The length of the chick was measured by stretching it along a graduated ruler and recording the length from the tip of the beak to the terminal middle toe, excluding the nail (Hill, 2001). Navel condition was recorded using a scoring system; 1 indicates a closed and clean navel, 2 indicates the presence of a string, a discolored button, or an open navel < 2 mm, and 3 indicates a black button or open navel > 2 mm (Molenaar, et al., 2010). The live body weight of each chick in all groups was recorded at DOH, D7, and D14, and then body weight gain **(BWG)** was calculated. Mortality was recorded throughout the experiment.

### Residual yolk and intestinal length measurement

On DOH, seven chicks per group were euthanized by CO_2_ asphyxiation. Chicks were dissected and the yolk sac were removed. The residual yolk was weighed and calculated relatively to live body weight. Similarly, on day 7 of age, seven chicks per group were euthanized and dissected, the entire small intestine was removed, and the lengths of the individual small intestinal segments were measured. Based on preliminary screening, intestinal sections from selected treatment groups were used for histology evaluation and immune cell isolation, and blood was collected for immune cell isolations.

### Histomorphometry of small intestine

At D7 of age, segments of the small intestine from 0 mg, 1 mg, and 25 mg groups were collected in 10% (v/v) formalin (4 samples/intestinal segment/group). These groups were selected for intestinal histomorphometry based on their hatchability levels. Then samples were processed and stained with hematoxylin and eosin staining. For each sample, three cross-sections were prepared per slide. Three images were captured for each sample, and within each image, three intact villi and their corresponding crypts were selected for measurements. Slide images were captured by All-in-One Fluorescence Microscope BZ-X810 (Keyence Corporation of America, IL, USA), and villus height (VH) and crypt depth (CD) were measured using Image J software (v 0.5.8) (National Institute of Health, MD, USA). VH to CD ratios were calculated.

### Isolation of peripheral blood mononuclear cells

At D7 of age, approximately 5 mL of blood was collected immediately after euthanizing birds (4 birds/group) in a 15 mL tube containing EDTA (Growcells, CA, USA). The isolation of PBMCs was performed as per the previously described protocol (Sharma, et al., 2025), with slight modifications. In brief, blood was diluted 1:1 with PBS and layered over 4 mL of Histopaqueࣨ−1077 (Sigma-Aldrich Co., Dorset, UK) and then centrifuged at 800 ×  g for 20 minutes. The buffy coat layer containing PBMCs was collected in 5 mL of staining buffer and centrifuged at 400 × *g* for 5 minutes. The PBMCs pellet was resuspended in staining buffer, and live cells were counted using the trypan blue exclusion method.

### Isolation of intraepithelial lymphocytes from ileum

At D7 of age, the ileum was harvested from eight birds/group, and IELs isolation was performed using a previously established protocol in chicken (Majeed, et al., 2024). In brief, ileum segments were flushed with PBS, cut longitudinally to remove intestinal contents, and cut into 1 cm fragments. The ileum fragments sections were agitated in media (PBS, 5% chicken serum, 2 mM DTT, and 2 mM EDTA) at 150 rpm, 37 °C for 45 minutes. The IELs fraction was enriched from the supernatant using a discontinuous 40/70% Percoll density gradient (Cytiva, Marlborough, MA, USA). The recovered cells were washed and resuspended in staining buffer. Cells were counted using the trypan blue exclusion method.

### Flow cytometry

For this part, we focus on the cellularity analysis of the 0 and 1 mg bmOPN groups as the latter group displayed the highest hatchability. An aliquot of 1 × 10^6^ live cells (PBMCs and IELs) was stained with fluorochrome-conjugated anti-chicken CD45 SPRD (LT40), CD4 PE-CY7 (CT-4), CD3 AF547 (CT-3), TCRγδ FITC (TCR-1), CD8α AF700 (CT-8), and CD8β PE (EP42) antibodies (SouthernBiotech, Brimingham, Al, USA). Cells were acquired on NorthernLights^TM^ flow cytometry (Cytek, Fremont, CA, USA). Fluorescence compensation was performed using single stained controls for each fluorochrome, with unstained cells used to assess autofluorescence. The gating of immune cell populations was performed using a previously established strategy (Majeed, et al., 2024). The frequency of cells was analyzed using FlowJo™ 10.10.0 (BD Biosciences, NJ, USA) software, and data were reported as the absolute cell numbers.

### Statistical analysis

Statistical analysis for all the parameters was performed using GraphPad Prism v10.0.3 software. All variables were assessed for normality using the Shapiro-Wilk test, and variables with *p* > 0.05 were considered normally distributed. Variables that did not follow a normal distribution (non‑parametric data) were analyzed using the Kruskal-Wallis test or the Mann-Whitney U test, as appropriate. One-way ANOVA was used to analyze data for body weight, BWG, residual yolk percentage, intestinal length, and histomorphometry, followed by the Tukey HSD test for mean comparison. Hatchability data were analyzed using a Generalized Linear Mixed Model, with treatments as the fixed effect and independent experiment (block) as the random effect, followed by the Tukey HSD test for mean comparison. Kruskal-Wallis test was used to analyze data for the hock condition, followed by the Dunn test for pairwise comparison. The Mann-Whitney U test was used to analyze data for flow cytometry. The results were expressed as mean ± standard error of the mean **(SEM)**. The level of significance was set at *p* < 0.05. A single bird was considered the experimental unit for all analyses.

## Results

### Hatchability

*In-ovo* administration of varying levels of bmOPN at ED 18 significantly increased egg hatchability (*p* = 0.005), with no significant block effect. Moreover, a significant linear dose-dependent trend was observed (*p* = 0.006), indicating that increasing bmOPN dose improve hatchability up to 25 mg bmOPN dose (Table 1). Both 1 mg and 25 mg groups exhibited the highest hatchability rates (93.6% and 93.9%, respectively), followed by the 10 mg groups (90.7%). In contrast, the administration of 50 mg bmOPN had a detrimental effect, as demonstrated by the reduction in hatchability to 80.3%. Furthermore, the administration of 0 mg and 0.1 mg bmOPN had comparable hatchability rates.

**Table 1:** Effect of *in-ovo* administration of bmOPN on hatchability, chick quality, and residual yolk weight.

	Treatments						
Measurements (DOH)	0 mg	0.1 mg	1 mg	10 mg	25 mg	50 mg	p value
Hatchability (%)	85.7 ± 5.4^bc^	83.9 ± 6.8^bc^	93.6 ± 3.9^a^	90.7 ± 3.9^ab^	93.9 ± 2.8^a^	80.3 ± 7.2^c^	0.005
Chick Length (cm)	17.79 ± 0.09^a^	17.53 ± 0.13^ab^	17.26 ± 0.09^bc^	17.11 ± 0.1^c^	17.01 ± 0.08^c^	17.67 ± 0.08^a^	<0.0001
Naval Score	84.56^b^	82.93^b^	102.9^ab^	107.5^ab^	121.8^a^	86.55^b^	0.0006
Residual Yolk Weight (%)	15.62 ± 0.94^a^	14.48 ± 0.64^ab^	13.45 ± 0.55^ab^	12.83 ± 0.83^ab^	11.76 ± 0.42^b^	13.78 ± 1.07^ab^	0.04

Specific pathogenic free layer’s fertile eggs were administered with varying concentration of bovine milk derived Osteopontin *in-ovo* on day 18 of embryonation. On day of hatch, hatchability was recorded, chick length was measured, navel of each chick was inspected to determine naval score, and residual yolk weight. was recorded. Naval score is presented as rank score means. Data are presented as mean ± SEM. Values with no common superscript differ significantly (*p* < 0.05).

### Chick quality

On the DOH, chicks in the 0 mg and 50 mg bmOPN exhibited significantly greater body length compared to those in the 1 mg, 10 mg, and 25 mg bmOPN groups, with the latter two groups showing the shortest chick lengths (Table 1). The chick length in the 0.1 mg groups was comparable to that of the 1 mg group, as well as the 0 and 50 mg groups. Furthermore, on the DOH, the birds in the 25 mg bmOPN group had significantly higher rank scores, indicating poorer naval conditions (Table 1), whereas no significant differences in naval scores were observed between the other treatments. All chicks in all treatments had normal hocks with no signs of redness (data not shown).

### Residual yolk weight

On DOH, *in-ovo* administration of bmOPN decreased the relative residual yolk weights (%) in a dose-dependent manner up to 25 mg (Table 1). However, only chicks in the 25 mg bmOPN group showed significantly lower residual yolk weight (%) compared to the 0 mg group.

### Body weight and body weight gain

*In-ovo* administration of bmOPN at doses of 1, 10, 25, and 50 mg significantly increased the body weight of hatched chicks compared to the 0.1 mg bmOPN group, but not the 0 mg control group (Table 2). This pattern in body weights persisted through day 7 of age. Interestingly, by day 14, chicks in the 1 mg and 10 mg groups showed the highest body weights.

**Table 2:** Effect of *in-ovo* administration of bmOPN on body weight and body weight gain.

	Treatments						
Measurements	0 mg	0.1 mg	1 mg	10 mg	25 mg	50 mg	p value
Body Weight (g)							
DOH	43.61 ± 0.78^ab^	42.04 ± 0.46^b^	44.73 ± 0.68^a^	44.68 ± 0.51^a^	45.7 ± 0.62^a^	44.81 ± 0.49^a^	0.001
Day 7	88.24 ± 1.25^a^	80.39 ± 1.46^b^	89.03 ± 1.18^a^	89.73 ± 1.3^a^	86.39 ± 2.0^a^	87.68 ± 1.1^a^	0.0002
Day 14	152.5 ± 3.85^c^	148.2 ± 3.25^c^	163.2 ± 3.32^ab^	166 ± 2.95^a^	150.9 ± 5.58^bc^	155.9 ± 3.49^bc^	0.01
Body Weight Gain (g)							
Day (0-7)	44.59 ± 1.01^a^	38.35 ± 1.42^b^	44.31 ± 1.09^a^	45.05 ± 1.26^a^	41.86 ± 1.6^ab^	42.89 ± 0.91^ab^	0.003
Day (0-14)	109.7 ± 3.53^bc^	106.1 ± 3.58^c^	118.6 ± 3.0^ab^	120.8 ± 2.76^a^	108.2 ± 4.62^bc^	111.4 ± 3.4^bc^	0.03

Specific pathogenic free layer’s fertile eggs were administered with varying concentration of bovine milk derived Osteopontin *in-ovo* on day 18 of embryonation. Body weight. of each chick was recorded on day of hatch as well as on day 7 and day 14 of age. Data are presented as mean ± SEM. Values with no common superscript differ significantly (*p* < 0.05).

The 10 mg bmOPN group exhibited the greatest body weight gain (BWG) on day 7, significantly higher than the 0.1 mg group (Table 2). By day 14, the 10 mg group, followed by the 1 mg group, demonstrated the highest BWG, while the 0.1 mg group had the lowest. Notably, birds receiving 10 mg bmOPN gained significantly more weight than all other groups. Collectively, *in-ovo* administration of bmOPN at 1–10 mg appears to have beneficial effects on body weight and BWG, whereas the 0.1 mg dose exerts a detrimental effect.

### Intestinal length and histomorphometry

**Fig. 1:**
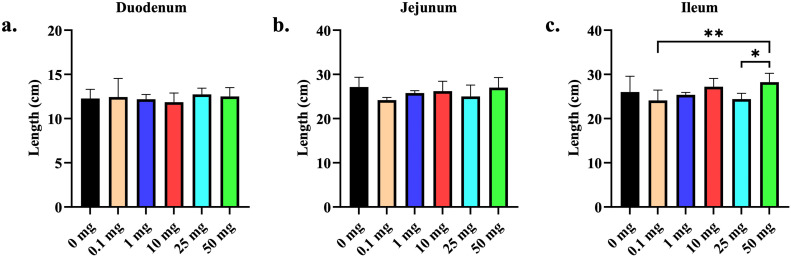
Effect of *in-ovo* administration of bmOPN on length of Duodenum, Jejunum, and Ileum. Specific pathogenic free layer’s fertile eggs were administered with varying concentration of bovine milk derived Osteopontin *in-ovo* on day 18 of embryonation. On day 7 of age, birds were euthanized and then length of different segments of intestine was measured. (a) Length of Duodenum. (b) Length of Jejunum. (c) Length of Ileum (*p* = 0.004). Data are presented as mean ± SEM. (*P < 0.05; **P < 0.01; ***P < 0.001; ****P < 0.0001).

**Fig. 2:**
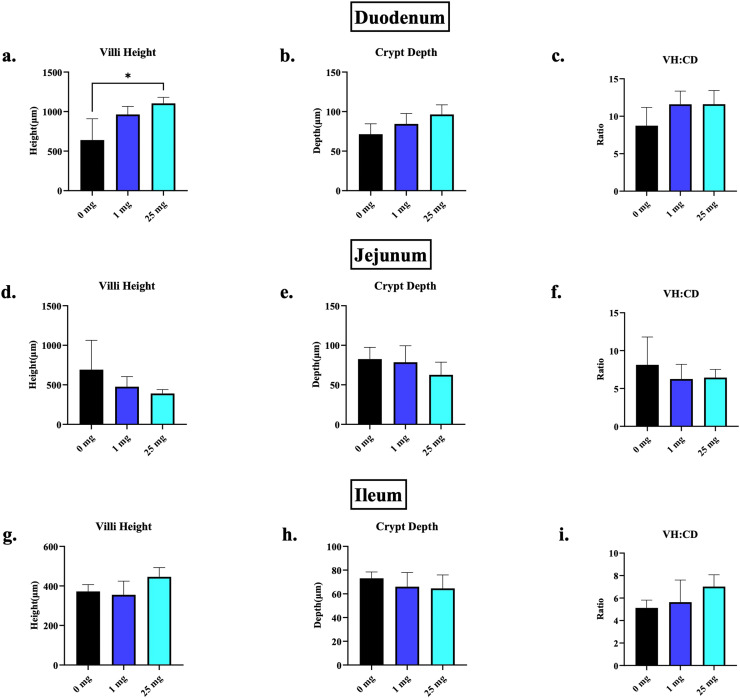
Effect of *in-ovo* administration of bmOPN on histomorphometry of Duodenum, Jejunum, and Ileum. Specific pathogenic free layer’s fertile eggs were administered with varying concentration of bovine milk derived Osteopontin *in-ovo* on day 18 of embryonation. On day 7 of age, birds were euthanized, and small segment of duodenum, jejunum, and ileum were collected in formalin, which was later used for histomorphometry (VH, CD, and VH:CD). (a) VH of Duodenum (*p* = 0.01). (b) CD of Duodenum. (c) VH:CD of Duodenum. (d) VH of Jejunum. (e) CD of Jejunum. (f) VH:CD of Jejunum. (g) VH of Ileum. (h) CD of Ileum. (i) VH:CD of Ileum. Data are presented as mean ± SEM. (*P < 0.05; **P < 0.01; ***P < 0.001; ****P < 0.0001).

At D7 of age, no differences were observed in the length of the duodenum and jejunum across all the treatment groups (Fig. 1). However, the chicks in the 50 mg bmOPN group had a significantly greater ileal length than those in the 0.1 mg and 25 mg bmOPN groups. Meanwhile, the birds in the 25 mg bmOPN group had significantly higher duodenal VH compared to those in the 0 mg group, but not the 1 mg group, while there were no differences in CD and VH:CD of the duodenum between the treatment groups (Fig. 2a). Furthermore, no differences in the jejunal and ileal VH, CD, and VH:CD were noted between the treatment groups (Fig. 2b and 2c).

### PBMCs analysis

**Fig. 3:**
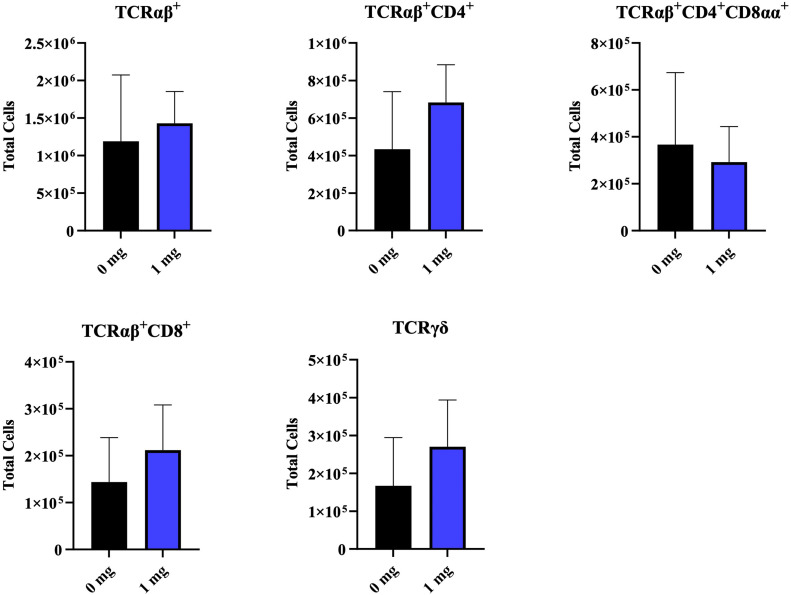
Effect of *in-ovo* administration of bmOPN on PBMCs. Specific pathogenic free layer’s fertile eggs were administered with varying concentration of bovine milk derived Osteopontin *in-ovo* on day 18 of embryonation. On day 7 of age, blood was collected from the birds for isolation of PBMCs and immune cells of interest was acquired using flowcytometry. Data are presented as mean ± SEM. (*P < 0.05; **P < 0.01; ***P < 0.001; ****P < 0.0001).

The administration of 1 mg bmOPN had no significant effect on the number of TCRαβ^+^cells or their subsets (TCRαβ^+^CD4^+^, TCRαβ^+^CD4^+^CD8α^+^, and TCRαβ^+^CD8^+^) as well as TCRγδ cells in the blood at D7 of age (Fig. 3).

### Intraepithelial lymphocytes analysis in ileum

**Fig. 4:**
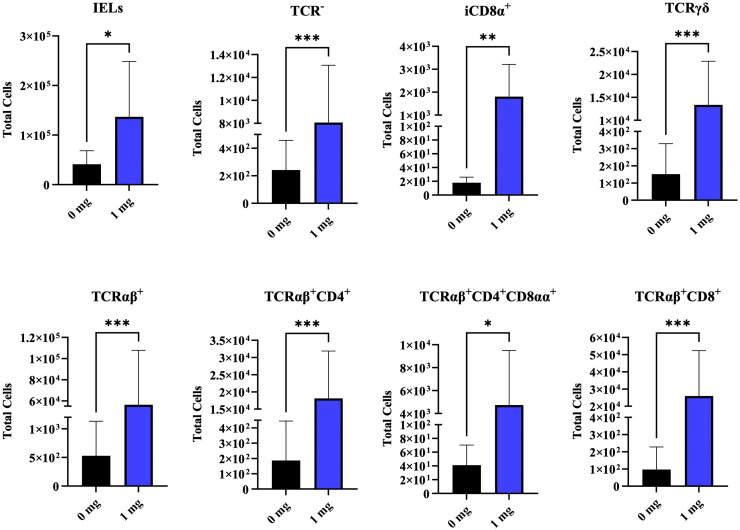
Effect of *in-ovo* administration of bmOPN on IELs. Specific pathogenic free layer’s fertile eggs were administered with varying concentration of bovine milk derived Osteopontin *in-ovo* on day 18 of embryonation. On day 7 of age, a segment of ileum was collected for isolation of IELs, and immune cells of interest was acquired using flowcytometry. Data are presented as mean ± SEM. (*P < 0.05; **P < 0.01; ***P < 0.001; ****P < 0.0001).

At D7 of age, *in-ovo* administration of 1 mg bmOPN significantly increased the total number of IEL subsets in the ileum (Fig. 4). Specifically, there was an elevated number of TCRαβ^+^ cells (TCRαβ^+^CD4^+^, TCRαβ^+^CD4CD8αα^+^ and TCRαβ^+^CD8^+^ subsets), TCRγδ, TCR^-^, and iCD8α^+^ cells.

## Discussion

For decades, antibiotic growth promoters **(AGPs)** have been routinely used in the poultry industry to promote faster growth, improve feed efficiency, and enhance overall health (Elwinger, et al., 1998). However, restrictions on AGP usage imposed by European Union, the USA, and Canada have been associated with the re-emergence of certain enteric diseases, such as necrotic enteritis **(NE)**, prompting extensive research into alternative strategies for disease prevention and control (Emami and Dalloul, 2021; Feng, et al., 2022; Hughes, et al., 2008; Martin, et al., 2015). For instance, research on AGP alternatives, such as probiotics, prebiotics, synbiotics, phytochemicals, enzymes, and organic acids, has yielded potential strategies for NE intervention, although none have been as effective as AGPs (Emami and Dalloul, 2021; Shah, et al., 2025). Consequently, the primary objective of this study was to investigate the potential of *in-ovo* supplementation of bmOPN as an alternative to AGPs in hatchability, early growth, and intestinal development in chickens during homeostasis.

This study specifically investigated the effects of *in-ovo* administration of varying concentrations of bmOPN on key developmental parameters that serve as important indicators of growth and health in hatched chicks during homeostasis. *In-ovo* injection at ED 18-19 is a common method for mass vaccination (Gagic, et al., 1999) and for delivering early feeding with various nutrients or probiotics to promote intestinal development, microbial colonization and immune system maturation (Alizadeh, Astill, Alqazlan, Shojadoost, Taha-Abdelaziz, Bavananthasivam, Doost, Sedeghiisfahani and Sharif, 2022; Jha, Singh, Yadav, Berrocoso and Mishra, 2019; Taha-Abdelaziz, et al., 2018). While there is a perception that *in-ovo* injection may compromise hatchability if not performed correctly (Das, et al., 2021), several studies demonstrated that *in-ovo* delivery of nutrients or bioactive compounds, such as creatine monohydrate and probiotics, can instead improve hatchability and hatchling quality (Firman, et al., 2023; Gao, et al., 2024). Therefore, we first assessed the safety of in-ovo administration of different bmOPN doses in hatched chicks by evaluating their effects on hatchability, chick quality, and growth performance.

In general, our results show that *in-ovo* administration of bmOPN at doses 1–25 mg had no adverse effects on hatchability and chick quality. Indeed, hatchability increased by approximately 8% in the 1 mg and 25 mg groups compared to the control, reaching 93.6% and 93.9%, respectively. At these bmOPN doses, the late embryonic mortality was reduced. OPN is calcium-binding protein that modulates calcium absorption, transport and deposition, thereby influencing the embryonic bone mineralization (Gericke, et al., 2005; Steitz, et al., 2002). During egg incubation, the eggshell is the main calcium source for embryonic skeletal development, and insufficient calcium availability during the late incubation can compromise skeletal mineralization, delay piping and reduce hatchability (Halgrain, et al., 2022; Torres and Korver, 2018). Therefore, bmOPN sublimination on ED 18 may enhance calcium mobilization, leading to increase hatchability. Chick quality serves as a key indicator of embryonic development, nutrient utilization, and post-hatch performance (Meijerhof, 2005). We observed decrease in chick length and residual yolk percentage, along with an increase in navel score up to 25 mg bmOPN. A well-closed navel is an important quality trait, as an open or stringy navel can serve as an entry point for pathogens, predisposing chicks to yolk sac infections and early mortality (Meijerhof, 2005). Although the 25 mg group exhibited a poorer navel condition compared to other groups, no morbidity or mortality was observed throughout the experiment, indicating that bmOPN administration did not adversely affect overall health.

In mammals, milk OPN is believed to stimulate the development of neonates (Aasmul-Olsen, Henriksen, Nguyen, Heckmann, Thymann, Sangild and Bering, 2021). In our study, *in-ovo* administration of 1–25 mg bmOPN increased chick hatch weights by approximately 1–2 g, coinciding with a reduction in residual yolk percentage, suggesting more efficient yolk utilization. Egg yolk contains various nutrients, with lipids serving as the primary source of energy (>90%) during late embryogenesis and the early post-hatch period, supporting body growth and development (Speake, et al., 1998). By day 14, chicks in the 1 mg and 10 mg bmOPN groups maintained higher body weights and body weight gains. Previous research has shown that chicks with higher hatch weights often exhibit early growth advantages, including enhanced gastrointestinal maturation and feed efficiency (Wolanski, et al., 2004). In our study, bmOPN treatment had variable effects on intestinal histomorphology on day 7. At day 7, bmOPN-treated chicks showed increased ileum length and duodenal villus height, indicating accelerated gastrointestinal maturation, expanded absorptive surface area, and enhanced digestive capacity, likely due to early exposure to bmOPN during the immediate post-hatch period (Svihus and Itani, 2019; Wolanski, Luiten, Meijerhof and Vereijken, 2004). The increase in ileum length may reflect OPN-mediated stimulation of intestinal epithelial proliferation, as OPN has been shown to enhance the expansion of goblet, Paneth, and enteroendocrine cell populations during early development in mice (Jiang, Lo, Prell and Lönnerdal, 2023), as well as promoting survival of IEL populations (Nazmi et al., 2020).

The role of OPN in maintaining IEL populations and intestinal homeostasis has been investigated in several studies (Ito, et al., 2017; Nazmi, Greer, Hoek, Piazuelo, Weitkamp and Olivares-Villagómez, 2020, 2019; Van Kaer, Algood, Singh, Parekh, Greer, Piazuelo, Weitkamp, Matta, Chaturvedi, Wilson and Olivares-Villagómez, 2014). Specifically, OPN-deficient mice displayed a reduced number of T-lineage IELs compared to wild-type mice, indicating that OPN enhances their survival and proliferation (Nazmi, Greer, Hoek, Piazuelo, Weitkamp and Olivares-Villagómez, 2020). Given that the immune system of newly hatched chicks is immature, we hypothesized that bmOPN might promote the development of immune cells. Our results were consistent with previous studies in mice (Nazmi, Greer, Hoek, Piazuelo, Weitkamp and Olivares-Villagómez, 2020), indicating that the main effect of OPN is confined to intestinal IEL and not systemic. On day 7, bmOPN administration increased the number of intestinal T-cell IELs (TCRαβ+ and TCRγδ+), as well as non-T IELs (including iCD8α+ cells), while no changes were observed in PBMC cells. These results indicate that *in-ovo* delivered bmOPN promotes the development of intestinal mucosal immunity and supports improved gut health in hatched chicks.

In conclusion, *in-ovo* administration of the bmOPN at doses of 1-25 mg was well tolerated, with no adverse effects on hatchability or chick quality. Instead, it improved chick performance, enhanced intestinal development, and strengthened mucosal immunity. Nevertheless, the optimal bmOPN dose for in ovo administration remains to be determined. Further studies are warranted to investigate the effects of bmOPN administered through different routes on intestinal function, bone development, and mucosal immune responses during inflammation in both broiler and layer chickens.

## CRediT authorship contribution statement

**Bikas R. Shah:** Writing – review & editing, Writing – original draft, Visualization, Software, Resources, Methodology, Investigation, Funding acquisition, Formal analysis, Data curation, Conceptualization. **Shuja Majeed:** Writing – review & editing, Methodology, Investigation, Data curation. **Nimra Khalid:** Writing – review & editing. **Pankaj Arora:** Writing – review & editing, Supervision. **Khaled Abdelaziz:** Writing – review & editing, Supervision. **Ali Nazmi:** Writing – review & editing, Validation, Supervision, Resources, Project administration, Investigation, Funding acquisition, Conceptualization.

## Disclosures

The authors declare the following financial interests/personal relationships which may be considered as potential competing interests:

Co-author employed by Arla Foods Ingredients Group P/S, Viby J, Denmark – P.A. If there are other authors, they declare that they have no known competing financial interests or personal relationships that could have appeared to influence the work reported in this paper.
